# A microfluidic device for studying chemotaxis mechanism of bacterial cancer targeting

**DOI:** 10.1038/s41598-018-24748-7

**Published:** 2018-04-23

**Authors:** Jing Song, Yu Zhang, Chengqian Zhang, Xiaohui Du, Zhe Guo, Yanbin Kuang, Yingyan Wang, Peng Wu, Kun Zou, Lijuan Zou, Jianxin Lv, Qi Wang

**Affiliations:** 1 Department of Respiratory Medicine, The Second Hospital, Dalian Medical University, Dalian, China; 2 Department of Radiotherapy, The Second Hospital, Dalian Medical University, Dalian, China; 3 Laboratory of Protein and Peptide Pharmaceuticals and Laboratory of Proteomics, Institute of Biophysics, Chinese Academy of Sciences, Beijing, China; 4 University of Chinese Academy of Sciences, Beijing, China; 5 Department of Scientific Research Center, The Second Hospital, Dalian Medical University, Dalian, China; 6 Department of Respiratory Medicine, The first Affiliated Hospital of Shantou University Medical College, Shantou, China; 7 Laboratory Center for Diagnostics, Dalian Medical University, Dalian, China; 8 Department of Radiotherapy, The First Hospital, Dalian Medical University, Dalian, China; 9 Key Laboratory of Medical Genetics, Wenzhou Medical University, Wenzhou, China

## Abstract

Bacterial cancer targeting may become an efficacious cancer therapy, but the mechanisms underlying bacterial specificity for cancer cells need to be explored prior to adopting it as a new clinical application. To characterize the mechanism of bacterial chemotactic preference towards cancer cells, we developed a microfluidic device for *in vitro* study. The device consists of a cell culture chamber on both sides of a central bacteria channel, with micro-channels used as barriers between them. The device, when used as model for lung cancer, was able to provide simultaneous three-dimensional co-culture of multiple cell lines in separate culture chambers, and when used as model for bacterial chemotaxis, established constant concentration gradients of biochemical compounds in a central channel by diffusion through micro-channels. Fluorescence intensity of green fluorescence protein (GFP)-encoding bacteria was used to measure bacterial taxis behavior due to established chemotactic gradients. Using this platform, we found that *Escherichia coli* (*E. coli*) clearly illustrated the preference for lung cancer cells (NCI-H460) which was attributed to biochemical factors secreted by carcinoma cells. Furthermore, by secretome analysis and validation experiments, clusterin (CLU) was found as a key regulator for the chemotaxis of *E. coli* in targeting lung cancer.

## Introduction

With increasing prevalence of cancer cases worldwide and current shortcomings of existing treatment methods such as chemotherapy, radiation therapy, surgery and transplantation, there is a call for the immediate development of novel cancer therapies. Due to the non-specificity of current therapies, recognition between cancer cells and normal cells is a challenging dilemma in cancer therapeutics. Bacterial-mediated cancer therapy is a novel alternative treatment currently under intensive study^1,2^. This therapy utilizes bacterial strains, which can have unique specificity for cancer cells, and the pathogenicity of bacteria themselves can be subdued by attenuation or other molecular techniques for genetic alteration^3,4^. During the last decade, development of numerous animal models has proved that bacteria play a role in tumor size reduction^5–7^. However, the exact mechanism of tumor degradation in bacterial-mediated cancer therapy is not fully understood and remains a challenging question for cancer therapeutics.

Possible explanations for the efficacy of bacterial therapy in the reduction of solid tumors include the ability of obligate anaerobes such as *Clostridia* and *Bifidobacteria* spp. to grow in necrotic regions with low oxygen, which is a unique and persistent characteristic of solid tumors^8,9^. In addition, facultative anaerobes like *Escherichia coli* (*E. coli*) and *Salmonella* sp. are competent enough to target tumors using more complex mechanisms including chemotaxis towards the biochemical elements secreted by carcinoma cells^10,11^. Meanwhile, previous studies suggest that *E. coli* strains possess variation in their ability to colonize in different tissues, e.g. *E. coli Nissle 1917* had little accumulation in spleen and liver as compared to that in tumors and in other tissues. While administration of *E. coli K-12* to mice bearing murine 4T1 breast carcinomas effectively stimulated an anti-tumor immune response and resulted in major reduction of pulmonary metastatic events^10^. In some previous reports, bacterial therapy has been employed *in vivo* using *E. coli* that has been attenuated to suppress toxic effects, but the attenuated strains expressed little activity as compared to the endogenous ones. However, in number of previous experiments relevant to microfluidic chip research, *E. coli* O157 showed the highest amount of activity^12–14^. So, in our study we employed a similar strain to analyze its potential for colonization in lung cancer cell lines.

Tumors express and secrete high concentrations of specific biochemical compounds which can be detected by various techniques of proteomics and computational biology^15,16^. However, biomolecular gradients and microfluidics have been recently applied for cancer diagnosis, cancer research and chemotaxis in cancer cell lines. Most of the adopted concentration gradients like gradient generators^17,18^ or Y-shaped channels^19,20^ are based on laminar flow and inadequate for maintaining a concentration gradient for biochemical molecules secreted by different cells due to its undistributed flow. Other gradient methods such as the plug-in-pond assay^21^ and self-assembled micro-particles^22^ used for cancer research are unable to be recruited for eukaryotic cell-based models where three-dimensional co-culturing of multiple cell types are required. Thus, it is essential to develop a platform that can offer a co-culture system for multiple cell types and establish a constant chemical concentration gradient for the biomolecules secreted by cells in order to analyze bacterial-specific chemotaxis.

In our research, we present a microfluidic device utilized to elucidate the chemotaxis mechanism of bacterial cancer targeting. A microfluidic platform is proposed to maintain an independent medium for three-dimensional multiple co-culture of multiple cell lines and to establish constant concentration gradients of biochemical molecules secreted by each cell type. The efficiency of this device was confirmed by examining the chemotaxis of *E. coli* towards 16HBE (normal human bronchial epithelial cells) and NCI-H460 (human large cell lung carcinoma) by using GFP-encoding bacteria. Furthermore, we developed the platform as a distinct tool to discover the diagnostic markers of lung cancer and define the role of Clusterin (CLU), a biochemical molecule contributing to the preferential bacterial chemotaxis towards NCI-H460 over 16HBE.

## Results

### Design and function of the microfluidic chip

**Figure 1:**
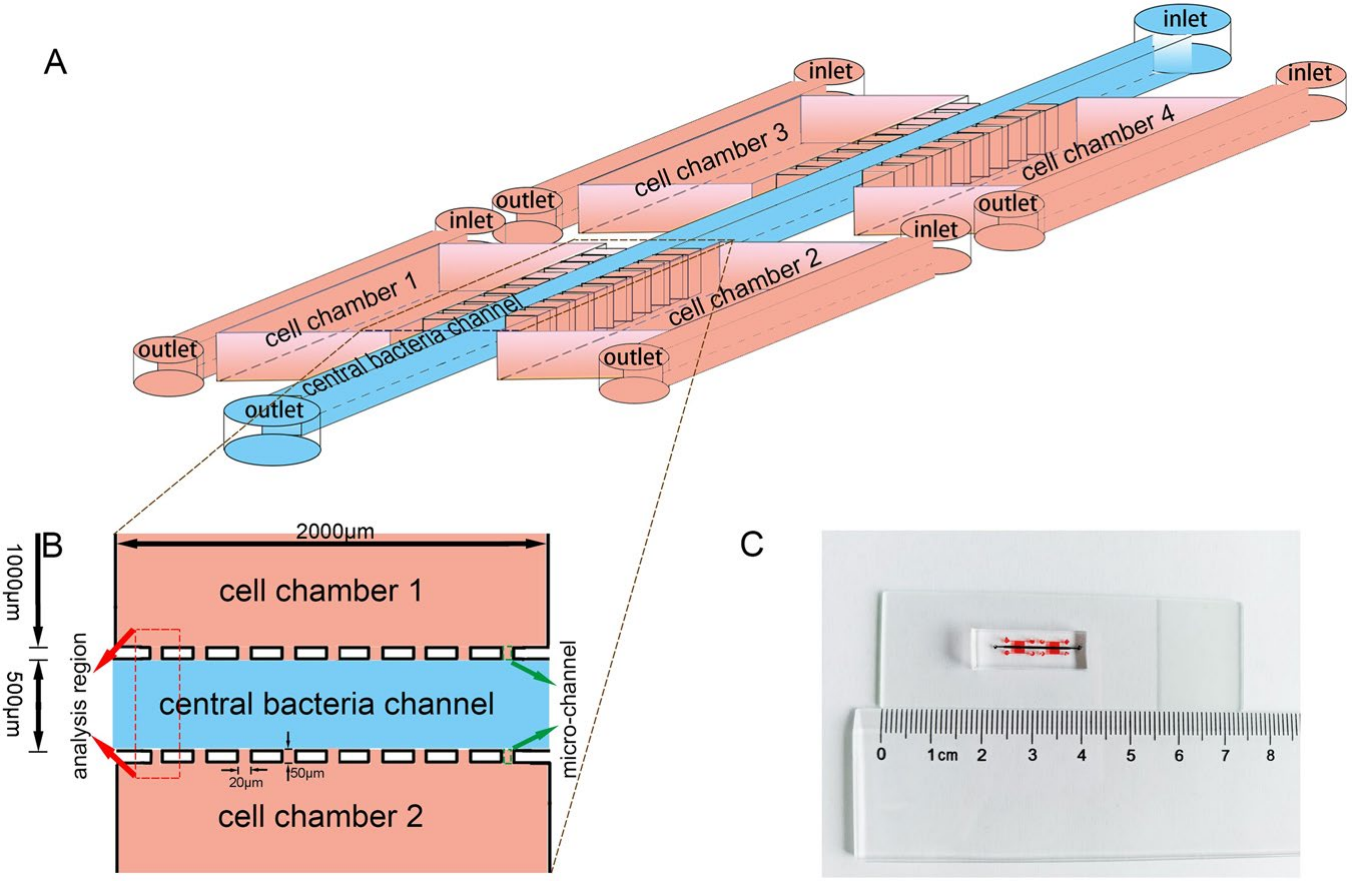
Design and illustration of the microfluidic device. (**A**) A schematic diagram of the device for studying bacterial chemotaxis mechanism. Microfluidic device comprised of four separated cell culture chambers, one central bacteria channel and micro-channels between them. (**B**) The analysis region was sited between two chambers of cell culture, where the preferential accumulation of bacteria could be quantified. Micro-channels were the barriers between the central bacteria channel and the cell chambers, and also acted as flow regulators. (**C**) A photographic overview of the integrated microfluidic device.

We designed an integrated microfluidic chip (Fig. 1) to analyze the chemotaxis mechanism of bacterial cancer targeting with certain requirements as follows: a separate culture media for multiple cell lines on a single device, stable concentration gradients of released biochemical molecules by the included cells, and a physical barrier between bacteria and the chambers containing cell cultures. The micro-channels (Fig. 1B) acted as the passive stop valves to stop the flow of liquid by the effect of capillary action, which were the essential components of the device. Micro-channels were made of Cultrex basement membrane extract (BME; R&D Systems, Minneapolis, MN, USA), which acted as a physical barrier between bacteria and cells to prevent entering bacteria into cell culture chambers.

**Figure 2:**
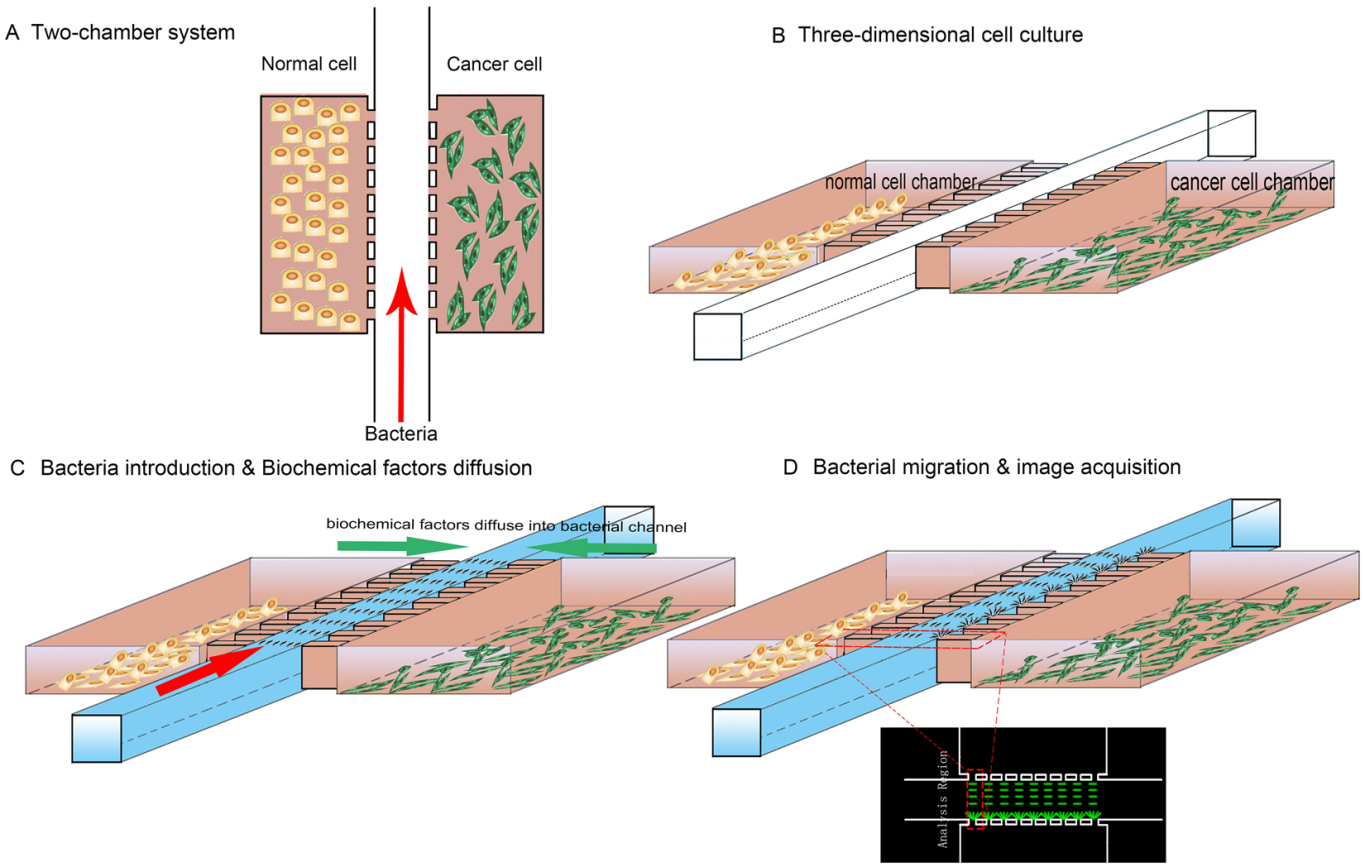
The schematic presentation of the experimental process of bacterial taxis. (**A**) The two-chamber device used for the experiments. (**B**) Normal and cancerous cells were cultivated for 48 hr in separate chambers. (**C**) GFP-encoding bacteria were injected into the central channel allowing interaction with the diffused biochemical factors from chambers of cell culture. (**D**) Preferential accumulation of bacteria was quantified in the analysis region by fluorescence intensity of GFP encoding bacteria.

The microchip design consisted of a cell culture chamber on each side of a central bacteria channel and micro-channels for separation between them (Fig. 2A). A mixture of normal cells was seeded in one of the cell chambers whereas cancer cells were inoculated into the other chamber, then the cultures were incubated for 48 hr to mimic three dimensional growth of cells *in vivo* (Fig. 2B). After the introduction of *E. coli* into the central bacteria channel, the liquids contacted both sides of the micro-channels, which established the connection between the cell culture chambers and central bacteria channel. The biochemical factors produced by cells in side chambers were able to diffuse into the central bacteria channel to form concentration gradients (Fig. 2C). Finally, due to the concentration gradients of biochemical factors in central bacteria channel, *E. coli* migrated towards the chamber containing cancer cells and images were visualized for the quantitative analysis (Fig. 2D).

### Quantification of concentration gradients

The microfluidic device was proposed to generate concentration gradients of biochemical molecules by diffusion through the barrier of micro-channels from the cell chambers into the bacteria channel; consequently, chemotaxis of bacteria can be induced. FITC-dextran, having molecular weight similar to the typical proteins secreted by the cultured cells, was used as model molecule to verify whether concentration gradients were established effectively via the barrier of micro-channels into the central channel of bacteria.

**Figure 3:**
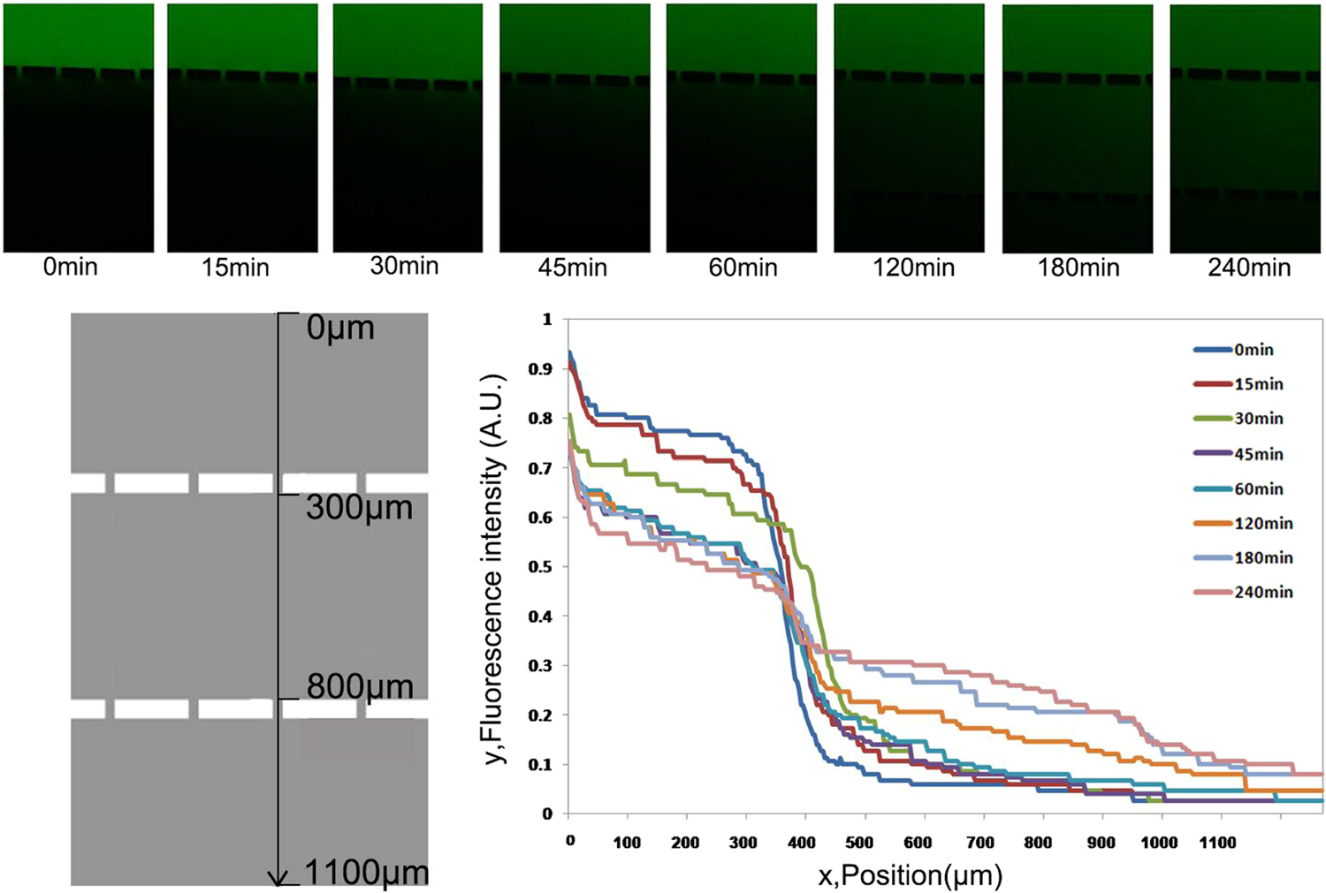
Quantification of concentration gradients across the device using FITC-dextran. Representative images of immunofluorescence of FITC-dextran with a concentration gradient in the central bacteria channel at different time points was showed. The measuring position of profiles of fluorescence was illustrated by the black arrow in the figure. The intensity of fluorescence was normalized as 1 at the corner of chamber of cell culture. When PBS was added in bacteria channel, an immediate diffusion of FITC-dextran from cell chamber to bacterial channel was observed and maintained for 4 hr with a stable circulation of dextran.

The visualization of fluorescence intensities due to diffusion of FITC was measured in the analysis regions of the bacterial central channel (Fig. 3). The molecules of FITC-dextran diffused into the central channel forming an immediate concentration gradient after the PBS was poured in the central bacterial channel. A nearly linear gradient was attained in approximately 1 hr and stabilized for over 4 hr (Fig. 3). Nevertheless, for previously designed chemotactic channels, continuous inflow was required to maintain concentration gradients in a channel filled by liquid. However, the three-dimensional cell culture used in this study was developed using BME and maintenance of a constant concentration gradients was demonstrated in the analysis region for at least 4 hr as configured.

### Analysis of chemotaxis mechanism of *E. coli*

**Figure 4:**
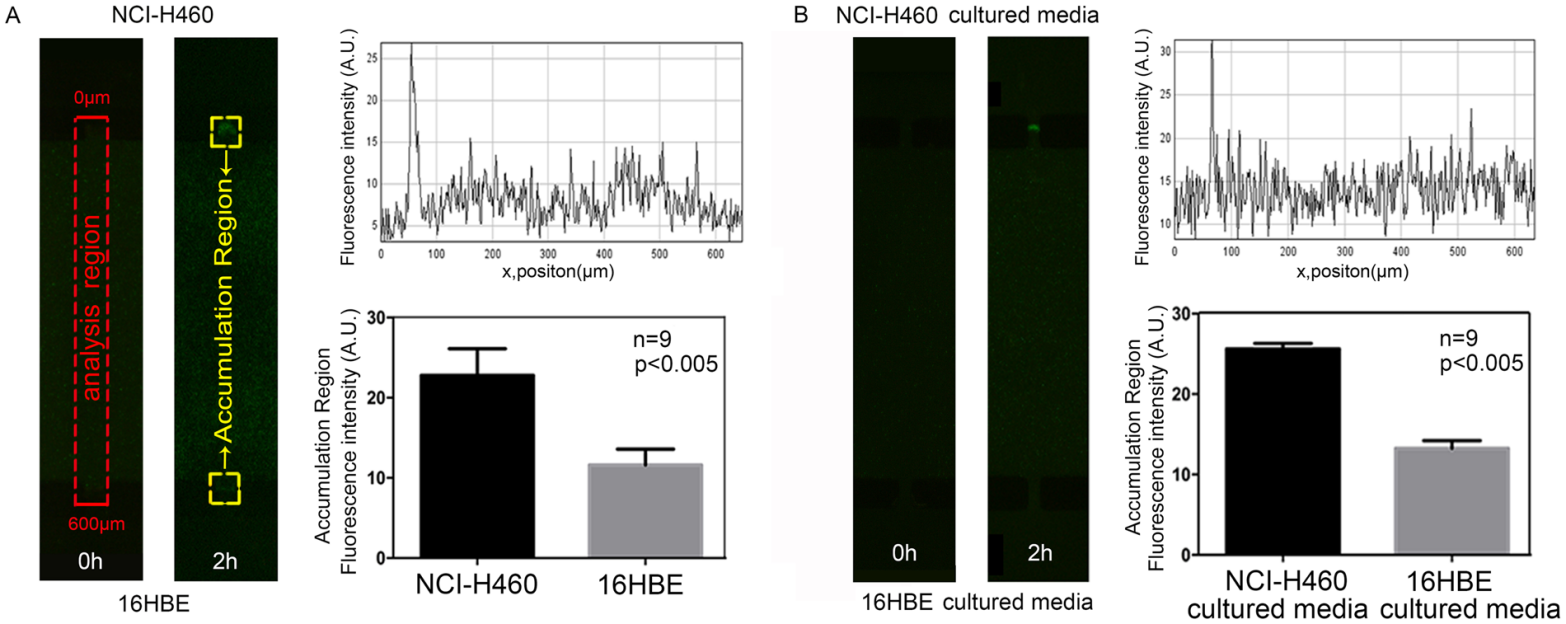
Analysis of quantitative bacterial chemotaxis. Bacterial chemotaxis was quantified by comparing the fluorescence intensity in the analysis region marked with red rectangle. Preferential accumulation of bacteria was compared by the fluorescence intensities in the two yellow marked rectangles of accumulation regions. (**A**) *E. coli* chemotaxis was firstly examined against NCI-H460 and 16HBE. The independent culture chambers were cultivated with cells, and the central channel was inoculated with bacteria. Significantly, higher preferential taxis of bacteria were illustrated towards cancerous cells as shown by the fluorescence images (left), and plots of intensities and average areal intensities of fluorescence (right). (**B**) The bacterial taxis towards cancerous cells, reasoned of biochemical molecules released by the cells, were examined by the preference of bacteria towards the medium used for cell cultures. The n = 9 represents 9 biological replicates, the error bars represent the standard deviation of means, and a difference of P < 0.05 was considered statistically significant.

Lung carcinoma cells (NCI-H460) and normal cells (16HBE) were cultured in separate chambers for 48 hr with serum-free media (Fig. S1). Almost 1 × 10^7^ cfu ml^−1^ of GFP-encoding *E. coli* diluted in PBS was introduced in the bacterial channel (Fig. 2C) and any difference of accumulations was monitored in the analysis regions (red-dotted rectangle as shown in Fig. 4A) using fluorescence microscope. The increased intensity of fluorescence in the accumulation regions (yellow-marked rectangle as shown in Fig. 4A) was interpreted as an evident affinity of *E. coli* towards NCI-H460 over 16HBE after 2 hr. Overall, in the accumulation regions, higher intensity depicted considerably increased accumulation of *E. coli* which was recorded two times more near to micro-channels of chamber containing NCI-H460 cells as compare to chamber containing 16HBE cells. The evidence of bacterial preference for NCI-H460 over 16HBE emphasized the presence of specific biochemical molecules released from NCI-H460 and diffused through the micro-channels that were able to attract bacteria.

A proposed cancer-targeting mechanism for facultative anaerobic bacteria included hypoxic germination, trophic factors and biochemical factors. We then sought to elucidate the chemotactic behavior of bacteria, induced not by hypoxic germination and trophic factors, but by cell-secreted biochemical factors. Dying cells may attract *E. coli* due to hypoxic germination, so we checked cell viability in order to eliminate the possibility of hypoxic germination in chemotactic preference of *E. coli* towards NCI-H460 cells. After 48 hr in culture chambers, both cells reached confluences of approximately 80%. As shown in Fig. S2, the survival rate of two cell lines was over 90% with no significant difference after 48 hr of bacterial inoculation.

Additionally, to elucidate the bacterial chemotactic preference was not caused by the trophic factors, RPMI-1640 medium without serum was tested. The medium and BME were injected into both chambers without cells for 48 hr to induce similar conditions. After 48 hr, central channels were filled with the bacteria and no preference was exhibited by bacteria for either chamber (Fig. S3).

### Confirmation of chemotaxis by *E. coli* due to secretion of biochemical molecules by cancer cells

In order to further prove whether secreted biochemical factors by cells caused chemotactic preference of bacteria towards cancer cells, NCI-H460, the chemotaxis of *E. coli* was determined by collecting the media of cells cultured for 48 hr in serum-free media. The obtained media were injected in the cell chambers with BME and after 1 hr, *E. coli* was introduced into the central channel. The bacteria showed similar behavior as in the condition of cell-culture, revealing distinct affinity for the medium of cancer cell culture (Fig. 4B). This finding depicted that chemotaxis of *E. coli* towards cancerous cells as compared to normal cells was persuaded by biochemical molecules secreted by cancerous cells.

### Bioinformatic study of possible biochemical candidates

**Figure 5:**
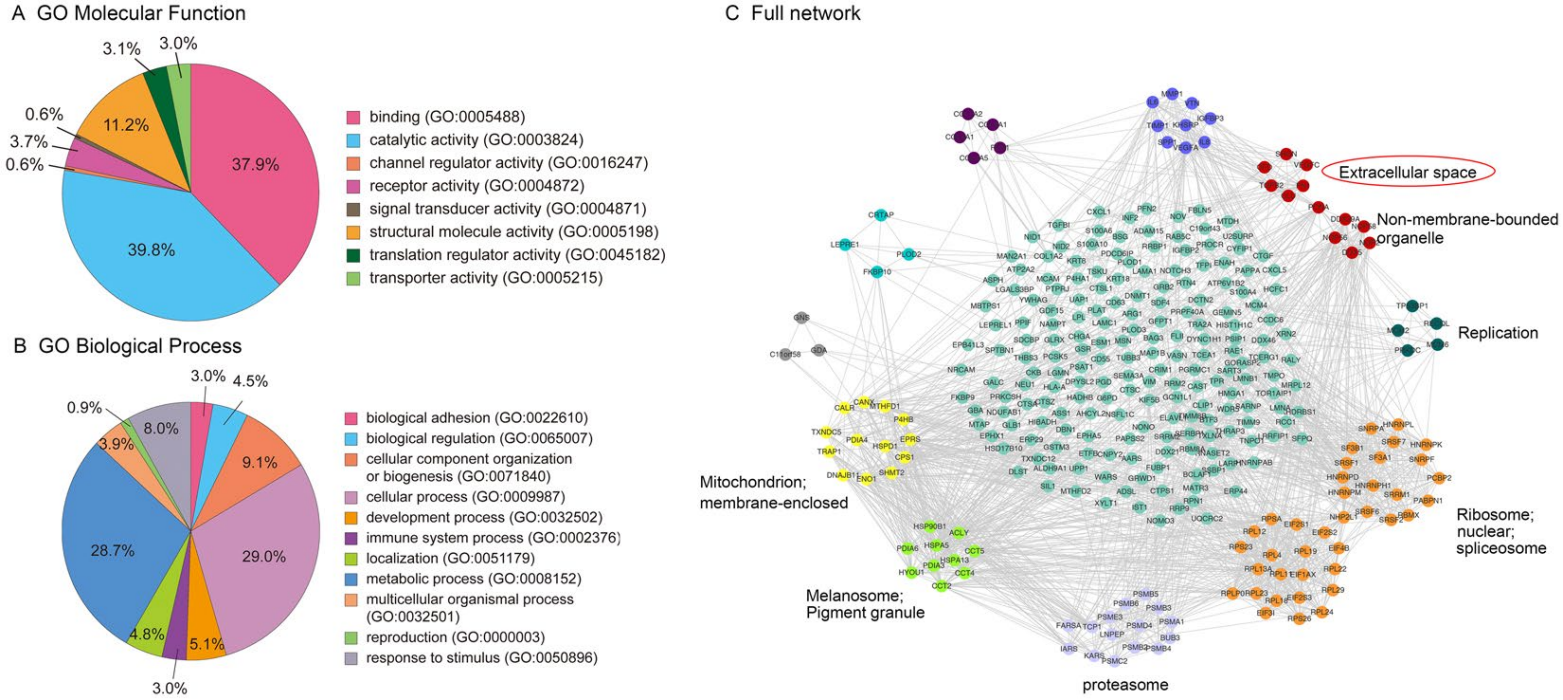
Bioinformatics analysis results of the proteomics of cell secretions. (**A**) Molecular function and biological processes analysis of the differential proteins were reported by PANTHER as characterized by gene ontology (GO). (**B**) The enrichment of organelles positioning analysis on the full network.

Secretions of NCI-H460 and 16HBE cell were detected by the LC-Q-Exactive mass spectrometer. Among 2313 overall identified proteins, 1055 were quantitative proteins; 486 differential proteins were observed (*P* ≤ 0.05 and the ratio value > 2). Data analysis by the public databases STRING protein-protein interaction networks, Cytoscape network visualization software and Mcode interaction network analysis, ten key network subsets were retrieved. GO ontology enrichment analysis and the enrichment of organelles analysis portrayed the organelle positioning, there were six network subsets which were related to the enrichment of the cells at different cellular compartments. Considering the analysis of protein in the secretions, we focused on proteins in extracellular space as possible biochemical candidates, which were clusterin (CLU), serglycin (SRGN) and transforming growth factor- β2 (TGFβ2) (Fig. 5).

### Identification of biochemical factors for chemotaxis

**Figure 6:**
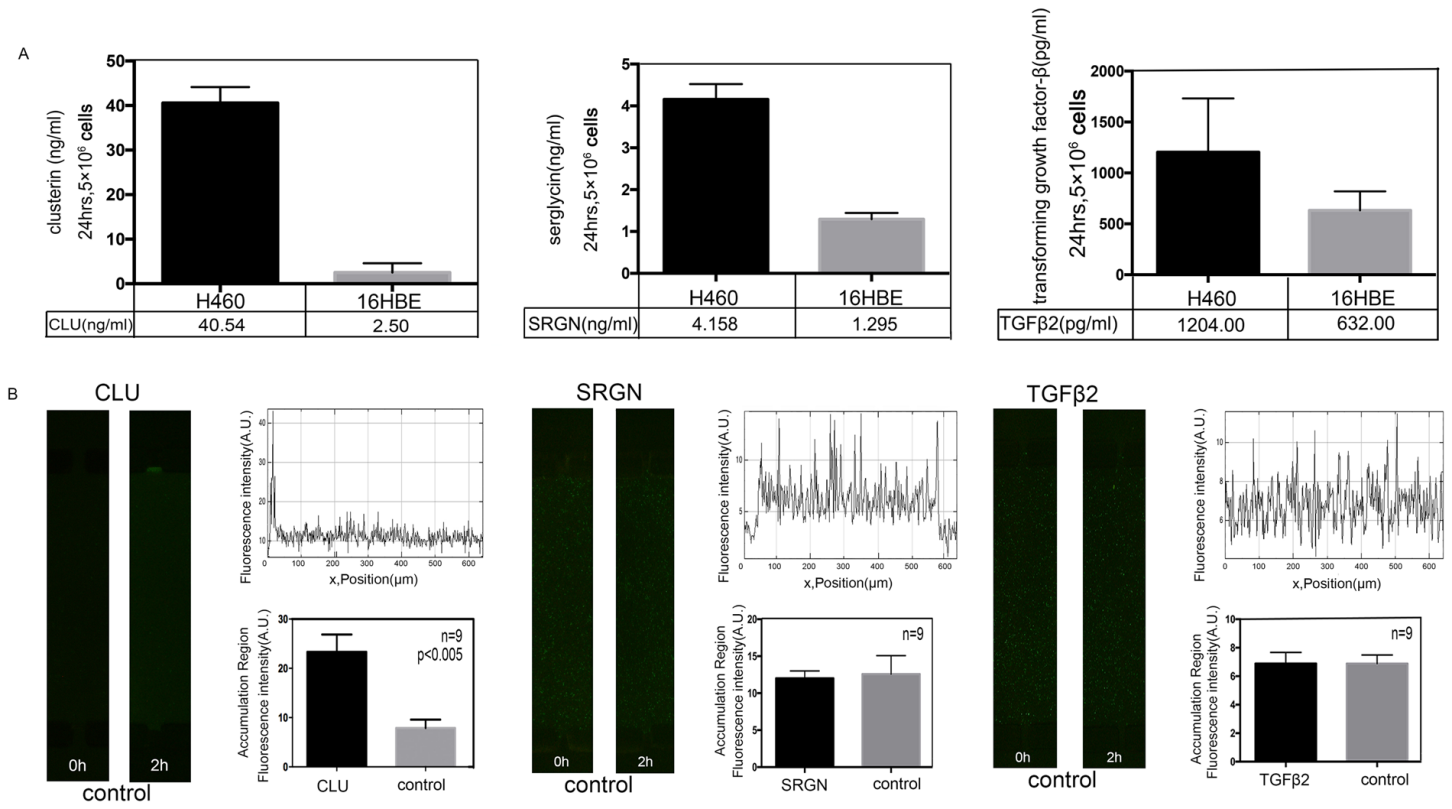
Verification of the possible biochemical candidates. (**A**) ELISA quantification of the possible biochemical candidates’ expression in normal and cancer cells revealed that NCI-H460 expressed more CLU, SRGN and TGFβ2 than 16HBE. (**B**) Quantitation of bacterial chemotaxis of the possible candidates. CLU, SRGN and TGFβ2 were tested as biochemical candidates that attracted *E. coli* towards NCI-H460. Clear preference was observed for CLU against control, but no significant chemotaxis was noticed for SRGN and TGFβ2. The n = 9 represents 9 biological replicates, the error bars represent the standard deviation of means, and a difference of P < 0.05 was considered statistically significant.

Based on the results of proteomics and bioinformatics analyses of cell secretions, we verified whether NCI-H460 cells expressed CLU, SRGN and TGFβ2 higher than 16HBE in similar conditions. The CLU, SRGN and TGFβ2 levels in cell-secreted media were calculated by ELISA. Quantification results (Fig. 6A) revealed that NCI-H460 expressed CLU, SRGN and TGFβ2 significantly higher, thus confirming them as possible biochemical factors in bacterial attraction.

To illuminate the biochemical candidates in the bacterial cancer targeting, CLU, SRGN and TGFβ2 were tested for their roles in bacterial accumulation. There were no significant differences observed in fluorescent intensities of SRGN and TGFβ2-supplemented media as compared to their control media (Fig. 6B). However, the preference analysis of *E. coli* for CLU-supplemented media against control media indicated evident chemotactic preference of bacteria at the CLU side. These findings provided evidence that CLU was of great importance to preferential chemotaxis responsible for the ability of *E. coli* to target lung cancer cells, NCI-H460.

## Discussion

As a leading cause of death in the world, cancer is still seemingly incurable. Due to such a large prevalence of cancer, it has been a popular and well-funded research area for decades. Moreover, inventions and discoveries are contributing to the understanding of cancer biology, diagnostics and treatment. Because of limitations in current therapies, bacterial-mediated cancer therapy has emerged as a key future prospect in cancer therapeutics and diagnostics due to its target specificity^2^. Bacteria have been reported to be able to degrade solid tumors, though the reason is not fully understood. Thus, it is necessary to elucidate the mechanisms of bacteria targeting cancer.

There are few studies on the mechanism of tumor-targeting bacteria based on microfluidic chips. Recently, a novel microfluidic chip was developed for *in vitro* studies where it was shown that concurrent multiple cell cultures maintained a constant gradient of biochemical molecules across the passage filled with collagen for analysis of preferential chemotaxis of *Salmonella typhimurium* towards cancerous hepatic cells as compared to normal cells, a design of our microfluidic device is strongly inspired by Hong *et al*.^23^. Moreover, a clear preference of *S. typhimurium* for cancer hepatocytes, HepG2 has been demonstrated. Where, alpha fetoprotein (AFP) a HepG2 specific protein, has been regarded as one of the key chemoattractants for *S. typhimurium* to target liver cancer cells.

In the present study, a multi-chambered microfluidic device was successfully designed to examine the chemotaxis preference of bacteria for carcinoma cells as compared to normal cells in a single test. The device consisted of a two-chamber system which provided independent co-culture environments. A continuous level of diffusion of biochemical molecules from chambers of cell culture via micro-channels and stable chemical concentration gradients in a central bacteria channel was maintained for a sufficient long time. The device designed for this study has following advantages to previously structured platforms: it modified the open cell chamber design to a relatively closed design, to prevent cell contamination; it allows the three dimensional culture of cells to mimic cell growth *in vivo*; it has a particular structural design of micro-channels to increase the detection accuracy of the device even after multiple repetitions of the same conditions; it quickly maintains a concentration gradient through micro-channels that enhances the efficiency of the device by saving time. Furthermore, *E. coli O157* employed for this microfluidic chip clearly illustrated the preference for biochemical factors secreted by lung cancer cells (NCI-H460).

Additionally, secretome analysis was performed to detect possible biochemical factors for chemotaxis. Generally, the serum free culture medium is used for cell culture in the secretory proteomics research. In serum-free culture, the cell growth is slow and autolysis may occur by which the cytoplasmic proteins may release but in minute quantities^24^. In the bioinformatics analysis, we focused on proteins of extracellular space as possible biochemical candidates (CLU, SRGN and TGFβ2). Parallel to our secretome analysis, proteomics profiling of benzo(a)pyrene (BaP)-induced lung cancer was conducted for screening of potential biomarkers and three proteins, neuropilin-2 (NRP2), clusterin (CLU) and A-kinase anchor protein 12 (AKAP12) were proposed as potential biomarkers of BaP-induced lung cancer^16^. Another study revealed that RGD-displaying *Salmonella* showed strong targeting efficiency which resulted in the regression of alphavbeta3-overexpressing cancer xenografts, and prolonged survival of mouse models of human breast cancer and human melanoma^25^. Finally, we clearly demonstrated the role of clustrin (CLU) released by lung cancer cells in taxis behavior of bacteria after a series of experiments and analysis.

The current study possessed certain limitations as the device developed was bacterial strain-specific and tissue-specific. Different bacterial strains and cells of other origins could be employed for other cancer models to elucidate the mechanisms of chemotaxis in bacterial cancer targeting. In addition, our device can be easily used to study cell communication where no intersection between cells and bacteria is required and then only the diffusion of soluble molecules is capably screened. In brief, applications of the present platform in various biological research fields are possible where the diffusion of several compounds between prokaryotic and eukaryotic cells need to be studied.

Additionally, our current data clearly illustrated the preference of *E. coli* for lung cancer cells (NCI-H460) which could be attributable to cancer cell-released biochemical factors. Analysis using proteomics and verification tests revealed clusterin (CLU) was one of the key biochemical molecules involved in the preferential chemotaxis of *E. coli* to target lung carcinoma cells. Although we investigated the lung cancer-targeting mechanisms by *E. coli*, further animal experiments and clinical studies are required to adopt it as a complete therapeutic approach for the development of cancer treatment. Moreover, the success of this method will depend on live, attenuated or genetically modified non-pathogenic bacterial species; the bacteria also can serve as ideal vectors for delivering therapeutic proteins to tumors as bacteria-based, gene-directed enzyme pro-drug therapy^26^.

## Materials and Methods

### Fabrication of the microfluidic chip

The microchip was designed comprising of two side cell culture chambers, a central bacteria channel in the middle and micro-channels for separation between them (Fig. 1A). The microchip platform was fabricated with polydimethylsiloxane (PDMS, Sylgard 184, Dow Chemical, Midland, MI, USA) using standard soft lithography methods. Wafers were coated with SU-8 photoresist (MicroChem Inc., Newton, MA, USA) to form film deposition of up to 100 µm. The PDMS, consisting of cross-linker and elastomer (1:10), was used on the photoresist for molding and baked at 80 °C for 12 hours, and the structure was then bonded to a glass slide with an oxygen plasma treatment and autoclaved prior to use in experiments^27^. The device was shown in Fig. 1C.

A barrier of micro-channels between central bacterial channel and cell culture chambers was constructed using BME (R&D Systems, Minneapolis, MN, USA). Liquid BME mixture was made of BME and cell suspension (1:1), FITC-dextran suspension or a protein suspension. Liquid BME mixture was introduced from the cell chamber inlet, firstly filled the cell culture chamber, then stopped when it reached the micro-channels because of capillary action, and lastly outflow from the cell chamber outlet. Then BME mixture incubated at 37 °C in a CO_2_ incubator to solidify to form physical barriers.

### Detection of diffused cell proteins in the central bacteria channel

Dextran-conjugated fluorescein isothiocyanate (FITC-dextran), with a molecular weight of 20 kDa (Invitrogen, Carlsbad, CA, USA), was diluted with PBS to a concentration of 60 mg ml^−1^. One side cell chamber was containing the BME mixture of FITC-dextran, and the other side cell chamber was containing BME mixture of PBS, and the central bacteria channel was filled with PBS to visualize the transport of biochemical molecules in the central bacteria channel. The FITC-dextran possessed equal molecular weight with typical biochemical factors secreted by the cells. The fluorescent particles were photographed in the central bacteria channel by camera with high resolution of 16.25 megapixel (Nikon DS-Ri2 camera, Nikon, Tokyo, Japan) under a fluorescence microscope (Nikon ECLIPS Ti inverted microscope, Nikon, Tokyo, Japan). The fluorescence intensity was quantified by ImageJ (National Institutes of Health, Bethesda, MD, USA)^28^.

### Cell preparation

Prior to the experiment, normal bronchial epithelial cells (16HBE) and human lung carcinoma cells (NCI-H460), provided by The Cell Bank of Type Culture Collection of the Chinese Academy of Sciences (Shanghai, China), were cultured in RPMI-1640 medium (Gibco, Long Islands, NY, USA) at 37 °C in a humidified atmosphere of 5% CO_2_. The cell culture media was also supplemented with 10% fetal bovine serum (FBS, Hyclone, Logan, UT, USA), penicillin (100 U ml^−1^) and streptomycin (100 μg ml^−1^). For the microfluidic experiment, 1 × 10^3^ concentration of 16HBE and NCI-H460 cells were seeded in each chamber (5 × 10^4^ cells cm^−2^) in serum-free RPMI-1640 medium and BME, and the incubation period was 48 hr to achieve stable growth and the secretion of biochemical molecules in chamber with cell culture.

### Bacterial preparation

*E. coli* O157 was provided by ATCC (Rockville, MD, USA) and transformed with the plasmid pGFPuv (Clontech Laboratories Inc., Takara Bio, Mountain View, CA, US). GFP-tagged *E. coli* was grown in Lysogeny Broth (LB; Oxoid, Altrincham, Cheshire, England) accompanied with 50 mg ml^−1^ of ampicillin (Solarbio, Beijing, China). Prior to use for microfluidic experiment, bacteria were grown with shaking at 37 °C for 18 hr to achieve exponential phase, and then fresh media was utilized for incubation of bacteria for 4 hr at 37 °C. The bacteria diluted in PBS were injected to fill the central bacterial channel at a concentration of 1 × 10^7^ cfu ml^−1^.

### Quantification of bacterial preference

The images of the analysis region were taken at 0 hr and 2 hr using a fluorescence microscope (Fig. S4). The bacterial chemotactic preference was quantified by measuring fluorescent intensities in the red-marked dotted-square as shown in Figs 1B and 2D. The difference in fluorescent intensity illustrated the change in number of bacteria accumulating at the chamber peripheries containing cell culture, which was quantified using ImageJ. A comparison of average intensities near all micro-channels in the yellow marked accumulation region (shown in Fig. 4A) was made for interpretation of results. The difference among groups was analyzed using t-test (GraphPad, La Jolla, CA, USA), and the *p* value less than 0.05 was considered statistically significant.

### Secretome analysis

Culture media of NCI-H460 and 16HBE cells were collected after incubation for 48 hr and proteomics analyses of the cell secretions were performed. Clarified supernatants were concentrated using 3 kDa ultrafiltration membranes for each sample (three biological replicates) and rinsed three times in 8 M urea. Protein yield was quantified by Bicinchoninic Acid (BCA) assay. An amount of 50 μg protein was treated with 10 mM of reducing agent 1, 4-dithiothreitol (DTT) at 37 °C for 1 hr, and subsequently alkylated with 40 mM iodoacetamide (IAA) for 30 min at room temperature in the dark. Protein samples were digested with urea by diluting 8 M urea solution to 1 M in the presence of 50 mM ammonium bicarbonate. Finally, trypsin was added at a 1:50 trypsin-to-protein mass ratio and incubated at 37 °C overnight. All the digested peptides samples were desalted on Oasis HLB SPE columns and then concentrated under vacuum^16^.

### Mass spectrometry analysis

Each sample of peptides was dissolved in 5 µl of 0.1% formic acid (FA), loaded onto an in-house packed C18 trap column (100 μm ID × 2 cm, 5 μm, ReproSil-Pur C18 AQ, Dr. Maisch GmbH, Germany) and separated on an in-house packed 20 cm analytical column with ReproSil-Pur C18-AQ 3 μm resin (Dr. Maisch GmbH, Germany). Peptide mixtures were analyzed by a Q-Exactive mass spectrometer (Thermo Fisher Scientific, Sunnyvale, CA, USA) coupled to an EASY-nLC 1000 HPLC system (Thermo Scientific). Two µg peptides were loaded onto column with buffer A (0.1% formic acid, 99.9% water) and flushed with 4–90% buffer B (0.1% formic acid in 99.9% acetonitrile) for a 120 min gradient time at constant flow rate of 280 nl/min, then followed by the gradient: 4–8% buffer B for 8 min; 8–22% buffer B for 77 min; 22–32% buffer B for 25 min; 32–95% buffer B for 1 min; 95% buffer B for 9 min. The mass spectrometer was operated in positive ion mode and data dependent acquisition strategy was applied. Full scan MS spectra (over 300 to 1600 m/z range) were acquired in the Orbitrap mass analyzer at a high resolution of 70,000 (m/z 200) with an automatic gain control (AGC) of 3e6 and a maximum fill time of 60 ms. The twenty most intense peaks were selected for fragmentation in the HCD collision cell with normalized collision energy 27%. Fragmentation spectra were acquired with a resolution of 17,500 at 200 m/z where maximum injection time was 80 ms, and a target value was 5e4. The precursor ions were isolated from a 2.0 m/z window. Peptides carrying positive charges 2 to 5 were selected for fragmentation and raw data were analyzed with Xcalibur v2.2 (Thermo Fisher Scientific, Sunnyvale, CA, USA).

### Data analysis

Raw MS data acquired (six files) were imported into MaxQuant version 1.2.2.5 for protein and peptide identification and quantitation using the iBAQ (intensity-based absolute quantification) method with false discovery rate, FDR < 0.01^29^. The MS/MS spectra were run against the human Uniprot FASTA database (92013 entries, updated on 10-2015). Trypsin was used as enzyme with up to two missed cleavages allowed. Cysteine carbamidomethylation was set as a fixed modification; N-acetylation of protein and methionine oxidation were selected as variable modifications. Quantification of peptides and proteins were performed with default settings and the feature “match between runs” was selected.

### Bioinformatics analysis

Protein-protein interaction (PPI) network was constructed using the public database STRING Version 10.0 (http://string-db.org/) for the proteins which were quantified at least twice in triplicate analysis for both NCI-H460 and 16HBE cell secretions with *P* ≤ 0.05 and the ratio value >2, indicating a significant difference. The Cytoscape software was employed to visualize the interaction network and analyzed with Mcode algorithm to calculate interconnected subgraphs of complex PPI network. In the end, Gene Ontology (GO) enrichment analysis and the enrichment of organelles positioning analysis were performed to describe the location of cellular components^16^. Considering proteins in secretions, we focused on proteins of extracellular space as possible biochemical candidates.

### Enzyme-linked immunosorbent assay

To confirm and quantify the level of possible biochemical candidates, the amount of CLU, SRGN and TGFβ2 in cell culture media was determined using Quantikine Human CLU basic kit (Boster systems, Wuhan, China), Quantikine SRGN basic kit (Westang systems, Shanghai, China) and Quantikine TGFβ2 basic kit (Boster systems, Wuhan, China). The cell culture media were prepared as previously described in the above experiments and stored at −80 °C before use. The media were mixed with reagents and incubated as per manufacturer guidelines (CLU Assay Diluent: 90 min, CLU basic Conjugate: 60 min, Substrate Solution: 30 min; SRGN Assay Diluent: 40 min, SRGN basic Conjugate: 20 min, Substrate Solution: 15 min; TGFβ2 Sample Active: 10 min, Assay Diluent: 90 min, TGFβ2 basic Conjugate: 60 min, Substrate Solution: 30 min) at room temperature. Optical density (OD) values were measured at 450 nm using a Microplate Reader (Thermo Scientific, Sunnyvale, CA, USA). The concentrations of possible biochemical candidates of each sample were calculated using a standard curve.

### Chemotaxis analysis of possible biochemical candidates

According to the proteomic analysis results, important candidates of lung cancer regulation, clusterin (CLU, 2 ug ml^−1^, proteintech, Wuhan, China), serglycin (SRGN, 2 ug ml^−1^, proteintech, Wuhan, China), transforming growth factor- β2 (TGFβ2, 2 ug ml^−1^, Abcam, England, UK) were examined for taxis of bacteria. The PBS and BME were poured in both of the cell culture chambers whereas candidate proteins were added only in one of the chambers.

## Electronic supplementary material

Supplementary Information
