# Supplementary material for: A microfluidic device for studying chemotaxis mechanism of bacterial cancer targeting
Source: Sci Rep. 2018 Apr 23;8:6394. doi: 10.1038/s41598-018-24748-7 (PMC5913277; doi:10.1038/s41598-018-24748-7)
Supplement: Supplementary file 1 — Supplementary Information [file 41598_2018_24748_MOESM1_ESM.pdf]

A microfluidic device for studying chemotaxis mechanism of bacterial cancer targeting

Running Title: A microfluidic system for studying chemotaxis

Author names and affiliations.

Jing Song [a 1], Yu Zhang [b 1], Chengqian Zhang [c d], Xiaohui Du [e], Zhe Guo [a f], Yanbin Kuang [a], Yingyan Wang [g], Peng Wu [c], Kun Zou [h], Lijuan Zou [b, *],Jianxin Lv [i, *], Qi Wang [a, *]

[a] Department of Respiratory Medicine, The Second Hospital, Dalian Medical University, Dalian, China.

[b] Department of Radiotherapy, The Second Hospital, Dalian Medical University, Dalian, China.

[c] Laboratory of Protein and Peptide Pharmaceuticals and Laboratory of Proteomics, Institute of Biophysics, Chinese Academy of Sciences, Beijing, China.

[d] University of Chinese Academy of Sciences, Beijing, China.

[e] Department of Scientific Research Center, The Second Hospital, Dalian Medical University, Dalian, China.

[f] Department of Respiratory Medicine, The first Affiliated Hospital of Shantou University Medical College, Shantou, China.

[g] Laboratory Center for Diagnostics, Dalian Medical University, Dalian, China.

[h] Department of Radiotherapy, The First Hospital, Dalian Medical University, Dalian, China.

[i] Key Laboratory of Medical Genetics, Wenzhou Medical University, Wenzhou, China.

* CORRESPONDENCE: Qi Wang: wqdlmu@163.com or wqdlmu@dmu.edu.cn, phone number: +86 0411 84671291, Fax: 041184671291; Jianxin Lv: jxlu313@163.com; Lijuan Zou: zoulijuan1963@sina.com.

[1] These authors contributed equally to this work.

Supplementary Information

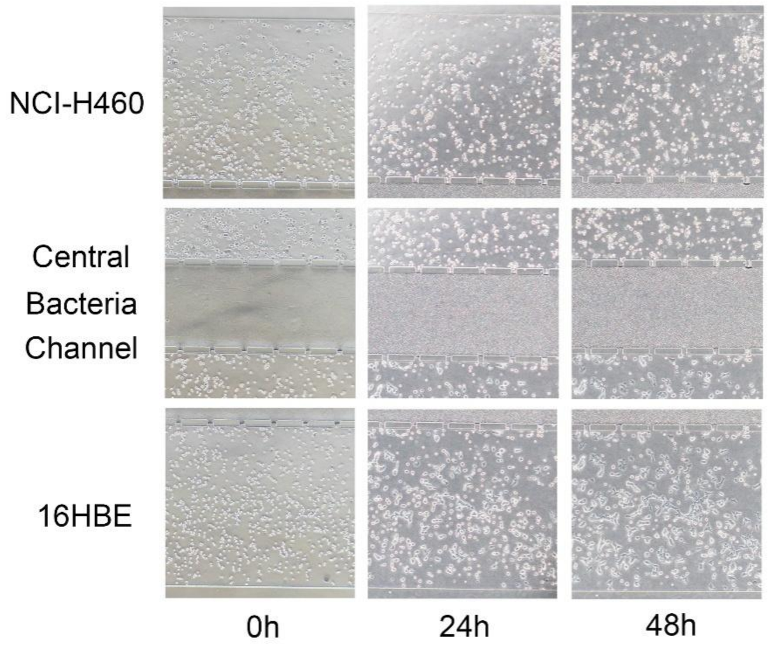

Supplemental Figure 1. Three-dimensional cell culture on the microfluidic platform. Three-dimensional culture status of NCI-H460 and 16HBE cells on the microfluidic platform were recorded by a microscope at different time intervals.

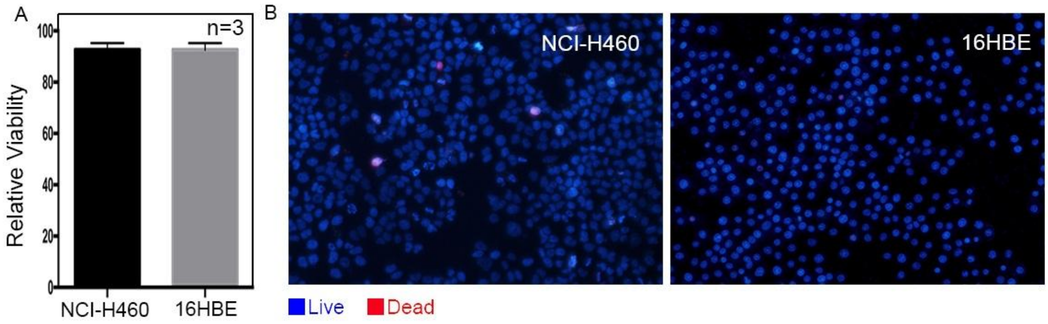

Supplemental Figure 2. Cell viability in isolated chambers. After 48 hr in culture chambers, both cell types attain confluency of approximately 80%. The viability of cells was evaluated to exclude the probability of dead cells to attract *E. coli* towards NCI-H460. For the viability assay, cells were thoroughly washed with PBS, before analyzing the survival rate using a viability test kit from Molecular Probes which utilized DAPI and PI to distinguish live and dead cells, respectively. The numbers of live and dead cells were counted from the images acquired with fluorescence microscope, and the ratio of live cells over total cells was normalized for comparison. After 48 hr of culture, more than 90% of cells of both types were alive with insignificant difference between two types of cells. The n=3 represents 3 biological replicates and the error bars represent the standard deviation of the mean.

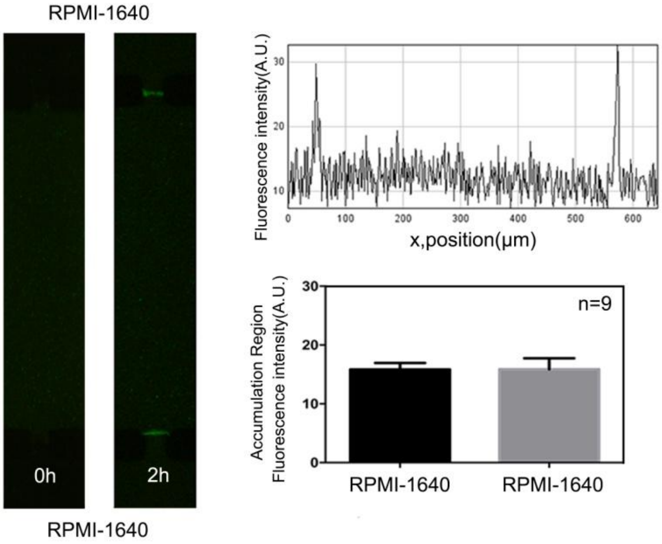

Supplemental Figure 3. Confirmation of the contribution of trophic factors in bacterial chemotaxis. RPMI-1640 media without serum was tested for this purpose. Media without serum was poured into two culture chambers for 48 hr to maintain a similar environment without cells. After 48hr, bacteria were inoculated into the central channel, and no preference was exhibited by the bacteria for either chamber and no significant difference was observed. The n=9 represents 9 biological replicates and the error bars represent the standard deviation of the mean.

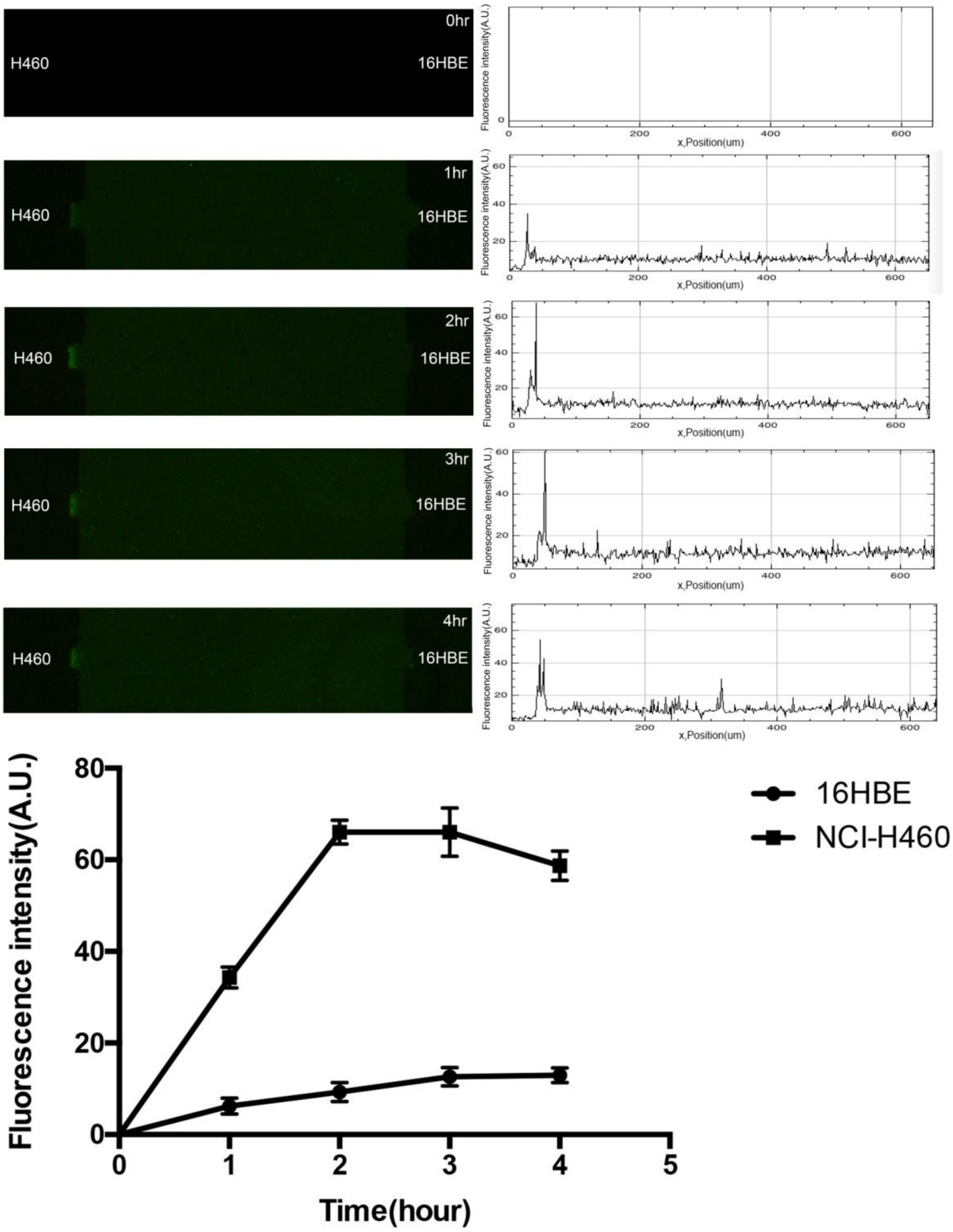

Supplemental Figure 4.Time points of bacterial taxis behavior against normal cell and lung cancer cell. Chemotaxis behaviors of bacteria against NCI-H460 lung cancer cell and 16HBE normal cell was observed for 4 hours. *E. coli* exhibited significant preference for the cancer cells.
